# The spatiotemporal distribution characteristics and influencing factors of ancient tombs in China: A study on the conservation of ancient tombs in China

**DOI:** 10.1371/journal.pone.0333485

**Published:** 2025-10-29

**Authors:** Quanbao Ma, Yujia Li, Zhen Yang, Xing Zhao, Can Li, Zi Shi, Zimu Li

**Affiliations:** 1 School of Architecture and Urban Planning, Beijing University of Civil Engineering and Architecture, Beijing, China; 2 Beijing Municipal Institute of Archaeology, Beijing, China; Universidad de Sevilla, SPAIN

## Abstract

Ancient tombs were valuable cultural heritage of China and possessed immeasurable significance. However, research on their spatiotemporal distribution and influencing factors was relatively absent. Based on the establishment of a national geographic information database of ancient tombs, this study employed ArcGIS and SPSS software to conduct both qualitative and quantitative analyses of the extant ancient tombs in China, with the aim of providing support for the protection of Chinese ancient tombs. The results showed that: ① The number of ancient tombs in the Qing Dynasty was the highest, while the number of ancient tombs from the Sui Dynasty accounted for the smallest proportion; ② Ancient tombs in different historical periods presented three distinct concentration areas around the Central Plains urban agglomeration, Chengdu-Chongqing urban agglomeration, and Guanzhong Plain urban agglomeration in China; ③ The focus of ancient tombs in different historical periods concentrated in the central region of China, with a recurring shift of the focus within the current provinces of Shaanxi, Shanxi, Henan, Hubei, and Chongqing; ④ The density of southern rice paddies (DL01) was negatively correlated with the distribution of ancient tombs in China, while the total GDP and population had a significant positive correlation with the distribution of ancient tombs. The significance of this study lies in systematically understanding the spatiotemporal distribution patterns and influencing factors of ancient tombs in China, in order to provide a theoretical basis for scientifically assessing risks, formulating effective protection plans, and guiding archaeological surveys and explorations.

## 1. Introduction

*The Principles for the Conservation of Heritage Sites in China* define heritage sites are the immovable physical remains that were created during the history of humankind and that have significance; they include archaeological sites and ruins, tombs, traditional architecture, cave temples, stone carvings, sculpture, inscriptions, stele, and petroglyphs, modern and contemporary sites and architecture, and historically and culturally famous cities, towns and villages together with their original components. Cultural landscapes and heritage routes and canals are also deemed to be heritage sites [1]. Among these, ancient tombs, as direct products of ancient human activities, embody the material civilization created by ancestors. They hold abundant historical information, such as the identity, social status, living customs, and religious beliefs of the tomb occupants, serving as critical material evidence for studying the social history of ancient China.

However, the protection of CATs currently faces challenges, including erosion from natural disasters [2], insufficient regulatory measures for protection, a shortage of specialized preservation professionals [3], conflicts between economic development and tomb preservation [4], and rampant tomb raiding activities [5]. Investigating the spatial distribution and influencing factors of CATs holds important implications for their conservation. Such research not only aids in uncovering the historical context and cultural significance of CATs and provides a scientific basis for their preservation but also facilitates the development of related academic disciplines and promotes the protection of this heritage.

Few studies have focused on the spatial distribution patterns and influencing factors of ancient tombs in China. Research on the spatiotemporal distribution characteristics and influencing factors of cultural relics and historic sites has primarily concentrated on ancient sites [6], ancient architecture [7], and cave temple [8]. The spatial distribution of these sites is influenced by both natural and human factors, and studies using qualitative and quantitative analysis have provided guidance for the protection of different types of cultural heritage. Research in the field of ancient tombs has mainly focused on three perspectives: exhibition and utilization [2,9], protection paths and strategies [3], and digital data collection [10]. Firstly, in terms of exhibition and utilization, scholars have analyzed targeted and appropriate approaches based on the current state, characteristics, and planning of CATs[2]. For non-popular CATs, such as Qin Gong Tomb No. 1, which are smaller in scale and attract fewer visitors, these can be jointly planned with nearby large-scale ancient sites as part of an integrated heritage display to improve regional industrial layout [9]. Secondly, regarding conservation approaches and strategies, relevant studies have focused on existing issues, cultural value, and resource conditions, aiming to enhance the conservation level of CATs by identifying influencing factors, leveraging environmental conditions, and improving legal frameworks [3,11,12]. Thirdly, in the area of digital data collection, photogrammetry technology has been applied to create 3D models of ancient tombs to analyze influencing factors. In a study on the digital data collection of ancient tombs in Guangxi based on close-range photogrammetry, experiments on the Kangxi King Mausoleum analyzed key factors affecting 3D modeling results, including weather, distance, and overlap rate. This research identified optimal modeling parameters for close-range photogrammetry, offering a methodological reference for the digitalization of cultural heritage [10]. Lastly, some studies [13] documented the archaeological excavation processes and results of ancient tombs, further analyzing the historical context of the tombs.

In terms of research methods, current studies on the spatiotemporal distribution characteristics and influencing factors primarily relied on the ArcGIS platform for various spatial and regression analyses [14–16]. Using the ArcGIS platform, techniques such as nearest neighbor index, centralization index, kernel density analysis, and elliptical analysis revealed that the spatial distribution of cultural relics and historical sites exhibited clustered patterns [17]. Building on this, methods like the geographical detector and geographically weighted regression were employed to determine the explanatory power of each influencing factor [18–20]. Additionally, some studies integrated data on cultural heritage protection units with factors such as topography, geomorphology, transportation, and rivers through overlay analysis and buffer analysis to further explore the correlations between influencing factors and spatial distribution [21–23].

In summary, whether in spatial analysis or quantitative research, studies focusing on CATs remain largely unexplored. To address the current gap in the research field concerning the distribution patterns of CATs and their complex influencing factors, this study adopted a comprehensive research methodology. The aim was to conduct a thorough and systematic analysis of CATs distributed across the country through data collection and analysis. The study not only focused on the numerical patterns, regional differentiation characteristics, spatiotemporal evolution, and influencing factors of ancient tomb distribution in China but also incorporated quantitative research.

In the implementation process, ArcGIS and SPSS were utilized. Spatial analysis techniques were employed to achieve precise and visually intuitive representations of the distribution of CATs, transforming potentially abstract and complex spatial data into comprehensible visual outputs. Meanwhile, regression analysis and other statistical methods were applied to quantify the multiple factors influencing ancient tomb distribution. This systematic and quantitative research approach provided a novel perspective and deeper understanding of the characteristics and evolutionary patterns of CATs. Furthermore, it offered robust data support and theoretical foundations for optimizing the protection strategies and inheritance mechanisms of historical and cultural heritage.

## 2. Data sources and research methods

### 2.1. Data sources

This study selected CATs, classified as immovable cultural relics across the country, as the research objects. The geospatial data of CATs used for analyzing spatiotemporal distribution characteristics were obtained from the Third National Cultural Relics Census. The census data underwent rigorous review and organization, resulting in reliable archival records and reports. These records document detailed information about CATs, including their chronology, locations, and preservation status, providing a dependable foundation for in-depth research.

The indicators used for analyzing influencing factors were categorized into two main groups. The first group focused on the socio-cultural aspects, encompassing two dimensions: socioeconomic factors and historical-cultural factors. The second group addressed natural geographic aspects, including six dimensions: river systems, climatic conditions, topography and landforms, road networks, vegetation coverage, and soil types.

Specifically, data on total population and GDP were sourced from the National Statistical Yearbook. Historical cultural figures were derived from local chronicles and biographies of historical personalities, providing important clues and contextual information for the study of CATs. The density of traditional villages was based on the lists of traditional villages from the first to sixth batches released by the Ministry of Housing and Urban-Rural Development. The density of administrative villages was obtained from the National Bureau of Statistics website. These datasets facilitated a macro-level understanding of the relationship between CATs distribution and human social activities, uncovering spatial patterns and primary influencing factors of CATs distribution. Temperature and precipitation data were retrieved from the China Meteorological Data Service Center, as these factors indirectly influenced the selection and evolution of human activity areas, thereby affecting the distribution of ancient tombs. Vegetation coverage, elevation, soil type, and river network density data were obtained from the Resource and Environment Science Data Center of the Chinese Academy of Sciences, while slope data were extracted and analyzed using ArcGIS. Runoff data were sourced from the National Tibetan Plateau Data Center. Since ancient tombs are mostly buried underground, the selection of tomb sites would have inevitably considered whether these natural factors could impact their long-term preservation, aiding in the revelation of spatial distribution characteristics. Road network density data were sourced from *Open Street Map*. This dataset reflected the intensity of human activity, influenced the preservation of CATs, and supported archaeological surveys and planning efforts.

However, since the data were recorded by multiple individuals, it inevitably contained duplicates, missing values, invalid entries, anomalies, and errors. To address this issue, the primary task was data cleaning, which involved removing duplicate and invalid information, filling in missing data, and correcting anomalies and errors. This process ensured that the raw data were transformed into a dataset with a uniform format and accurate content.

### 2.2. Research methods

#### 2.2.1. Kernel density estimation.

Kernel density analysis uses a kernel function to calculate the quantity per unit area based on point features, thereby reflecting the density characteristics of point features in geographic space. This method can visually represent the distribution and clustering characteristics of CATs in China [24]. Higher kernel density values indicate greater spatial clustering of CATs. The formula is as follows:


$$f_{n}(\begin{array}{ccc} x) & = & \frac{\text{1}}{nh}\sum_{i=\text{1}}^{n}k(\begin{array}{c} \frac{x-x_{i}}{h} \end{array}) \end{array}$$ (1)

In the formula, *n* represents the number of CATs in the study area in China, *h*(*h > 0*)denotes the bandwidth, which determines the smoothing level of the density estimate, *k*($\frac{x-x_{i}}{h}$) refers to the kernel function, which defines the shape of the influence of each point over the space,(*x-x*_*i*_)represents the distance between each estimated point (*x*) and the sample point (*x*_*i*_).

#### 2.2.2. Spatial autocorrelation.

The spatial autocorrelation analysis in this study was used to evaluate the degree of spatial correlation of CATs. By calculating the Moran’s Index, the spatial clustering characteristics of CATs in China were determined. The analysis primarily involved Global Moran’s I and Getis-Ord Gi statistics. The formulas are as follows:


$${\text{Moran}}^{\prime }\text{s I}=\frac{\sum_{i=\text{1}}^{n}\sum_{j=\text{1}}^{n}W_{ij}\;\left(X_{i}-\overset{―}{X}\right)(X_{j}-\overset{―}{X})}{S^{\text{2}}\sum_{i=\text{1}}^{n}\sum_{j=\text{1}}^{n}W_{ij}\;}\;$$ (2)

$$Gi^{*}=\frac{\sum_{j=\text{1}}^{N}Wij\cdot X_{j}-\overset{―}{X}\sum_{j=\text{1}}^{N}Wi_{j}}{\sqrt{\frac{\sum_{j=\text{1}}^{N}X_{j}^{\text{2}}}{N}-{\left(\overset{―}{X}\right)}^{\text{2}}}\cdot \sqrt{\frac{N\sum_{j=\text{1}}^{N}W_{ij}^{\text{2}}-\left(\sum_{j=\text{1}}^{N}W_{ij}\right)}{N-\text{1}}}},\forall j\neq i\;$$ (3)

In the formula, N represents the number of prefecture-level cities, $S^{\text{2}}$ denotes the variance of the data. $X_{i}$ and $X_{j}$ indicates the number of CATs in each prefecture-level unit. $\overset{―}{\text{X}}$ represents the mean number of CATs across all prefecture-level units. $W_{ij}$ refers to the spatial weight matrix representing the spatial relationship between regions (*i*) and (*j*). The values of ${\text{Moran}}^{\prime }\text{s I}$ range from −1–1. ${\text{Moran}}^{\prime }\text{s I}$>0 indicates a positive spatial correlation, meaning CATs exhibit spatial clustering characteristics; ${\text{Moran}}^{\prime }\text{s I}$<0 indicates a negative spatial correlation, meaning CATs exhibit spatial dispersion; ${\text{Moran}}^{\prime }\text{s I}$=0 indicates that the spatial distribution and location of the study object are random.

#### 2.2.3. Hotspot and coldspot analysis.

The local association index Getis-Ord Gi can identify the degree of spatial heterogeneity of elements at a local scale. In this study, the Getis-Ord Gi index was used to measure the clustering of high and low values of CATs in local spaces, revealing cold and hot spot areas in the spatial distribution. The formula is as follows:


$$G_{i}^{*}(d)=\frac{\sum_{j=\text{1}}^{n}w_{ij}(d)x_{j}}{\sum_{j=\text{1}}^{n}x_{j}}$$ (4)

In the formula, *d* represents the distance scale for each prefecture-level unit; *xj* refers to the observed value of ancient tombs in the (j)-th prefecture-level unit; *wij* denotes the spatial adjacency weight matrix value between the (i)-th and (j)-th units within the study area.

#### 2.2.4. Nearest neighbor index.

The Nearest Neighbor Distance is an indicator used to measure the degree of proximity of point features in geographic space [25]. In this study, the ancient tombs of China were taken as the research object, and the Nearest Neighbor Index was calculated at the national scale to reflect the proximity and dispersion of CATs in geographic space [17]. The formula is as follows:


$${\overset{↼}{r}}_{E}=\frac{\text{1}}{\text{2}\sqrt{n/A}},R=\frac{{\overset{↼}{r}}_{\text{1}}}{{\overset{↼}{r}}_{E}}$$ (5)

In the formula, *R* represents the Nearest Neighbor Index; *r*_*E*_ denotes the theoretical nearest neighbor distance; *n* is the number of CATs in the study area; *A* is the total area of the study region; *r*_*1*_ refers to the observed nearest neighbor distance. R < 1 indicates a clustered distribution of CATs; R = 1 indicates a random distribution of CATs; R > 1 indicates a uniform distribution of CATs.

#### 2.2.5. Standard deviation ellipse.

The Standard Deviation Ellipse (SDE) is a method used to measure central tendency, dispersion, and directional trends by determining the center, the angle of rotation, and the lengths of the X and Y axes [24]. The SDE provides an intuitive representation of the spatial distribution characteristics of CATs, including their center of gravity, directional orientation, and spatial extent [26]. The formula is as follows:


$$C=\frac{\text{1}}{n}(\begin{array}{cc} \sum_{i=\text{1}}^{n}{\overset{―}{x}}_{i}^{\text{2}} & \sum_{i=\text{1}}^{n}\overset{―}{x_{i}}\overset{―}{y_{i}}\\ \sum_{i=\text{1}}^{n}\overset{―}{x_{i}}\overset{―}{y_{i}} & \sum_{i=\text{1}}^{n}{\overset{―}{y}}_{i}^{\text{2}} \end{array}),\left\{ \begin{array}{c} \left(x_{i}-\overset{―}{x^{\prime }}\right)\\ \left(y_{i}-\overset{―}{y^{\prime }}\right) \end{array}\right.\;$$ (6)

In the formula, *X*_*i*_ and *Y*_*i*_ represent the coordinates of the (i)-th CATs, $\left(\overset{―}{x^{\prime }},\overset{―}{y^{\prime }}\right)$represent the mean center of the CATs, *n* represents the total number of CATs.

#### 2.2.6. Ridge regression.

Ridge Regression is a biased estimation regression method designed to address collinear data. Essentially, it optimizes the ordinary least squares (OLS) estimation by sacrificing unbiasedness to achieve more realistic and robust regression coefficient estimates. In this study, ridge regression was used to handle the multi-collinearity issues present in the analysis of CATs [27]. The formula is as follows:


$$\hat{\beta }(k)={\left(X^{T}X+kI\right)}^{-\text{1}}X^{T}Y$$ (7)

$$Y={\hat{\beta }}_{\text{0}}+{\hat{\beta }}_{\text{1}}X_{\text{1}}+{\hat{\beta }}_{\text{2}}X_{\text{2}}+\ldots +{\hat{\beta }}_{k}X_{k}$$ (8)

In the formula, $\hat{\beta }(k)$ represents the improved least squares regression parameters, k is the ridge parameter, $X^{T}$ represents the transpose of the independent variable matrix, I represents the identity matrix, Y represents the vector matrix of the dependent variable, ${\hat{\beta }}_{\text{0}}$ is the intercept term, ${\hat{\beta }}_{k}$ represents the standardized regression coefficient of the (k)-th influencing factor, the higher absolute value of indicates that this factor has a more impact on the spatial distribution or characteristics of CATs compared to other factors. The expression for the relative contribution rate is as follows:


$$C_{k}=\frac{\left|{\hat{\beta }}_{k}\right|}{\left|{\hat{\beta }}_{\text{1}}|+|{\hat{\beta }}_{\text{2}}|+\cdots +|{\hat{\beta }}_{k}\right|}$$ (9)

In the formula, $C_{k}$ represents the relative contribution rate of the (k)-th influencing factor on CATs; $\left|{\hat{\beta }}_{k}\right|$ represents the absolute value of the regression coefficient.

#### 2.2.7. Geographically weighted regression.

Geographically Weighted Regression (GWR) is a spatial analysis method commonly used in geography or related disciplines involving spatial analysis. It addresses the phenomenon of spatial non-stationarity by performing localized parameter estimations through assigning different spatial weights to neighboring units. While meeting the assumption of spatial dependency, GWR directly detects the heterogeneous effects of a certain factor across different spatial locations, outputting varying influence coefficients for each spatial unit. It serves as a classic model for analyzing the spatial heterogeneity of causal relationships. This study utilized GWR to explore the correlation between the number of ancient tombs and influencing factors across different municipal units [28]. The formula is as follows:


$$\begin{array}{c} y_{i}=\beta_{\text{0}}\left(u_{i},v_{i}\right)+\sum_{k=\text{1}}^{m}\beta_{k}\left(u_{i},v_{i}\right)X_{ik}+\varepsilon_{i} \end{array}$$ (10)

In the formula, The coordinates of the (i)-th sample is $\left(u_{i},{\;v}_{i}\right)$, $y_{i}$ represents the number of CATs at different spatial locations, $X_{ik}\;$ represents the observed value of the independent variable at spatial location $\left(u_{i},{\;v}_{i}\right)$, $\beta_{\text{0}}\left(u_{i},v_{i}\right)$ represents the constant term, $\varepsilon_{i}$represents the mutually independent random error term, $\beta_{k}\left(u_{i},{\;v}_{i}\right)$ represents the regression parameter of the (i)-th sample, its estimated value can be visualized on a map, allowing for a straightforward observation of the spatial distribution of the influencing factors’ impact on the distribution of CATs.

## 3. Results

### 3.1. Spatiotemporal distribution characteristics

#### 3.1.1. Temporal distribution characteristics.

**Fig 1:**
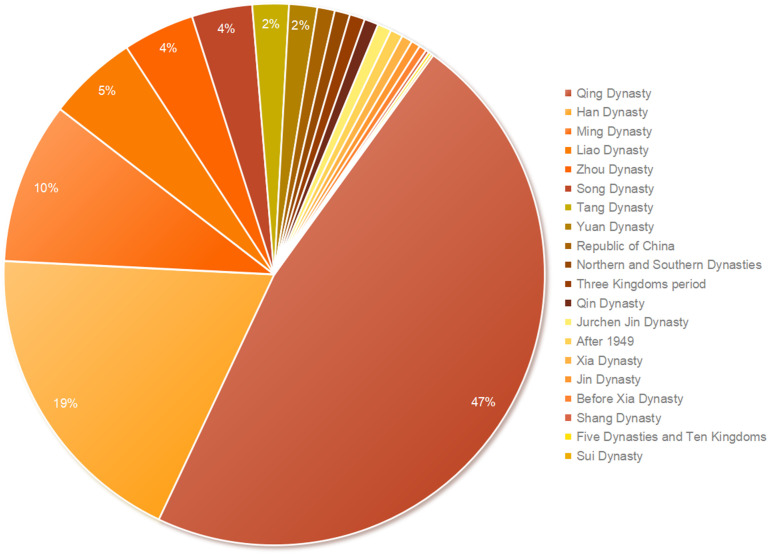
Percentage of the number of CATs in different historical periods.

The distribution of CATs in China across different historical periods exhibits significant variability, with the Qing Dynasty accounting for the largest proportion and the Sui Dynasty the smallest. Specifically, China’s historical timeline is divided into 20 periods based on dynasties. The number of CATs from the Qing Dynasty is far greater than those from other periods, comprising 47.010% of the total. The Han and Ming Dynasties also have relatively high proportions, accounting for 18.780% and 9.670%, respectively. The remaining periods contribute less, each representing less than 6% of the total. The Sui Dynasty has the fewest CATs, with only 0.130% of the total (**Fig 1** and **Table 1**).

**Table 1:** Statistics on the number and percentage of CATs in different historical periods.

Ranking of the number of CATs	Dynasty	Quantity	Percentage by dynasty
1	Qing Dynasty	44245	47.012%
2	Han Dynasty	17673	18.778%
3	Ming Dynasty	9104	9.673%
4	Liao Dynasty	5040	5.355%
5	Zhou Dynasty	4020	4.271%
6	Song Dynasty	3397	3.609%
7	Tang Dynasty	2049	2.177%
8	Yuan Dynasty	1581	1.680%
9	Republic of China	985	1.047%
10	Northern and Southern Dynasties	864	0.918%
11	Three Kingdoms period	860	0.914%
12	Qin Dynasty	794	0.844%
13	Jurchen Jin Dynasty	786	0.835%
14	After 1949	701	0.745%
15	Xia Dynasty	606	0.644%
16	Jin Dynasty	507	0.539%
17	Before Xia Dynasty	420	0.446%
18	Shang Dynasty	193	0.205%
19	Five Dynasties and Ten Kingdoms	164	0.174%
20	Sui Dynasty	126	0.134%

**Fig 2:**
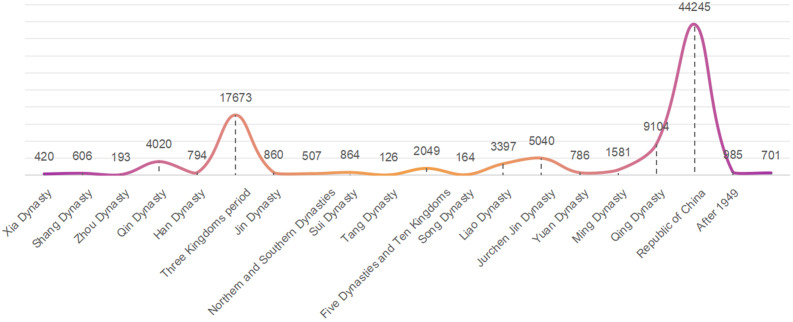
Changes in the number of CATs during different historical periods.

The changes in the number of CATs across different historical periods can be divided into four phases: fluctuating rise, stabilization, sharp increase, and decline.The first phase extended from the pre-Xia period to the Han Dynasty, during this phase, the number of CATs gradually increased with fluctuations. Notably, the number of CATs from the Zhou Dynasty showed a significant rise compared to earlier periods, although there was a slight decline during the Qin Dynasty. The second phase spanned from the Three Kingdoms period to the Liao and Jin Dynasties, this phase exhibited a relatively stable trend in the number of CATs, with slight increases during the Tang and Liao Dynasties. The third phase covered the period from the Yuan Dynasty to the Qing Dynasty, the number of CATs rose sharply during this phase, reaching its peak in the Qing Dynasty, which recorded the highest number of tombs among the 20 historical periods. The fourth phase began after Republic of China, the number of CATs in China entered a declining trend (**Fig 2**).

#### 3.1.2. Spatial distribution characteristics.

**Overall spatial distribution characteristics:** The spatial distribution density of CATs exhibited significant variations across different regions, with a national average density of 0.013 units/km². At the provincial, autonomous region, and municipality levels, the highest density was observed in Chongqing Municipality, with 0.188 units/km², while the lowest density was found in Tibet Autonomous Region, with less than 0.001 units/km². Provinces such as Hubei, Shaanxi, Sichuan, and Henan had relatively high densities, all exceeding 0.050 units/km². In contrast, Gansu Province, Shanghai Municipality, Jilin Province, Yunnan Province, Xinjiang Uighur Autonomous Region, Inner Mongolia Autonomous Region, Qinghai Province, and Heilongjiang Province had lower densities, all below 0.005 units/km². At the prefecture-level city scale, 126 city units, accounting for 38.890% of the total, had a tomb density of less than 0.060 units/km², while 94 city units, representing 29.010%, exhibited densities exceeding 0.240 units/km² (**Table 2**).

**Table 2:** Number and proportion of municipal units based on grave density classification of CATs.

Hierarchy	Density of CATs (units/km2)	Number of municipal units	Number of municipal units as a percentage	Municipal unit size(km^2^)	Percentage of municipal unit area
Extremely low	<0.060	126	38.89%	1102316.440	22.91%
Low	0.060-0.120	49	15.12%	1227688.420	25.51%
Medium	0.120-0.180	40	12.35%	719635.140	14.95%
High	0.180-0.240	15	4.63%	254387.130	5.29%
Extremely high	>0.240	94	29.01%	1508016.270	31.34%

**Fig 3:**
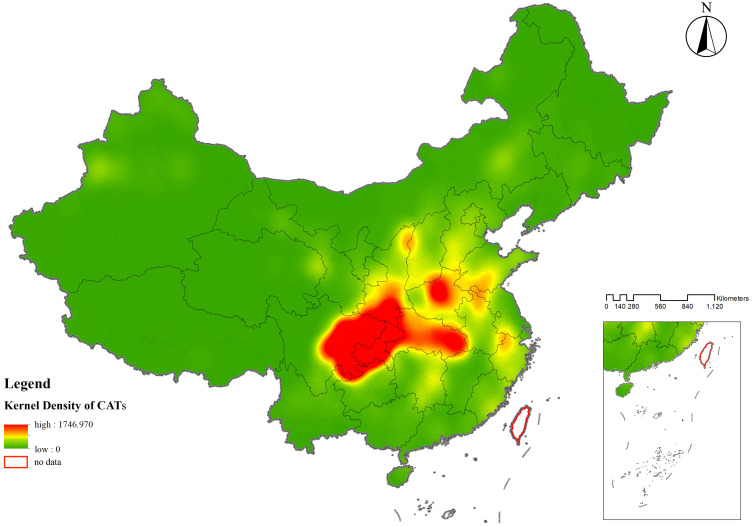
Kernel density analysis map of spatial distribution of CATs.

**Fig 4:**
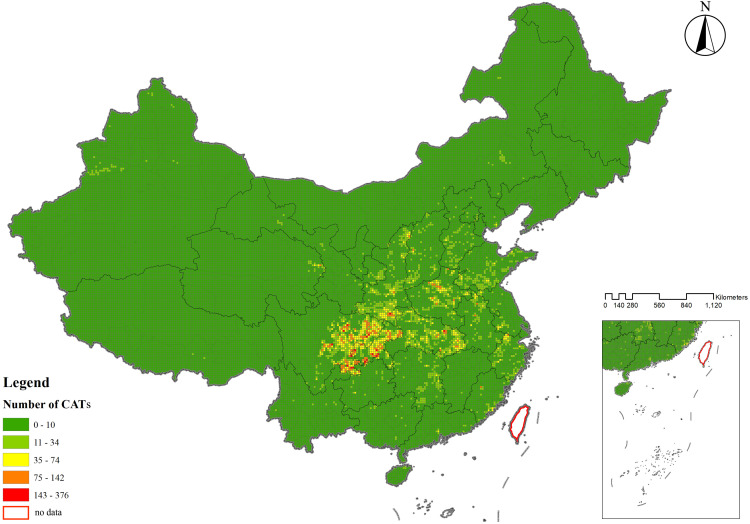
Fishing net analysis map of spatial distribution characteristics of CATs (20 km as a fishing net unit).

**Fig 5:**
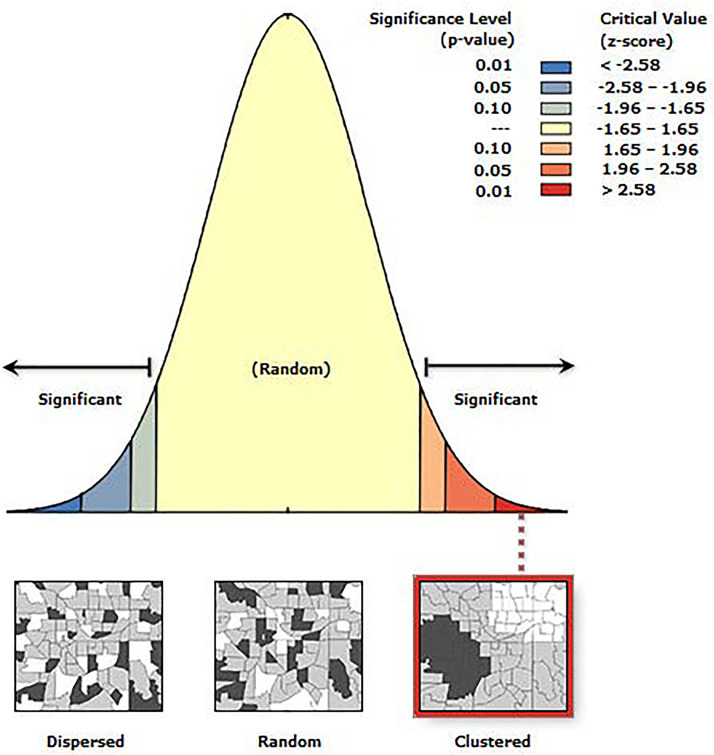
Global autocorrelation analysis of spatial distribution of CATs.

**Fig 6:**
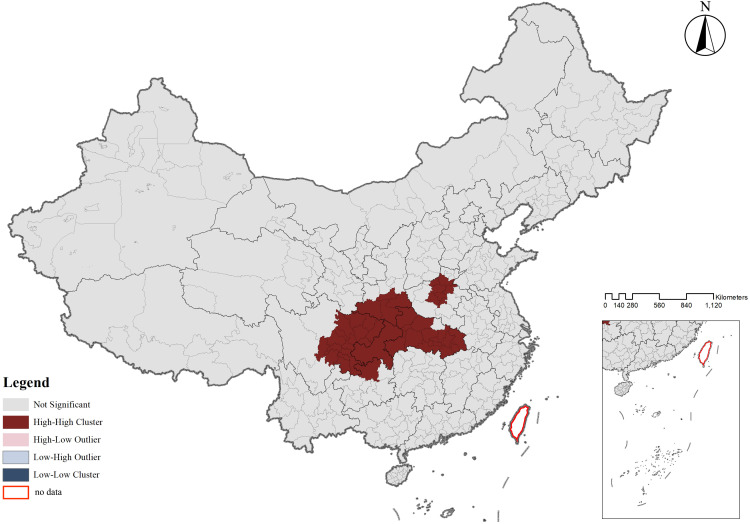
Plot of localized moran’s index (clustering and outlier analysis) for CATs.

The spatial distribution of CATs demonstrated a regional differentiation pattern, with the primary clustering area located in the Sichuan-Chongqing region and secondary clustering areas in northern Henan and eastern Hubei, showing a highly significant clustering tendency. To further evaluate whether the distribution of CATs exhibited a clustering or dispersal trend, as well as the intensity and significance of such a trend, geographic data of CATs were analyzed using kernel density analysis and grid analysis with a 20 km grid unit (**Fig 3** and **Fig 4**). Subsequently, a global spatial autocorrelation analysis was conducted on the geographic data of CATs. Using GIS computations combined with the Moran’s I index, the results indicated a “clustered” pattern, suggesting that the CATs spatially clustered to form hotspots or coldspots, rather than being randomly distributed. This implies that the spatial dependency of CATs was influenced by their proximity to neighboring geographic locations (**Fig 5**). To explore detailed relationships, a further analysis of the Local Moran’s I index was conducted, which identified abnormal values or clustering patterns. When the Local Moran’s I index was greater than 0, it indicated a positive spatial correlation, meaning neighboring features exhibited a “high-high” or “low-low” clustering trend. Conversely, when the index was less than 0, it indicated a negative spatial correlation, where neighboring features showed a “high-low” or “low-high” distribution trend. If the Local Moran’s I index approached 0, it suggested a random spatial distribution without any correlation. In analyzing the distribution data of CATs, 46 prefecture-level units, including Yuezhou, Guang’an, and Xi’an, were identified as “high-high” clusters. This indicated that these areas had relatively high densities of CATs, and this high density was spatially adjacent to other high-density areas, forming regions with significant clustering of CATs (**Fig 6**).

**Fig 7:**
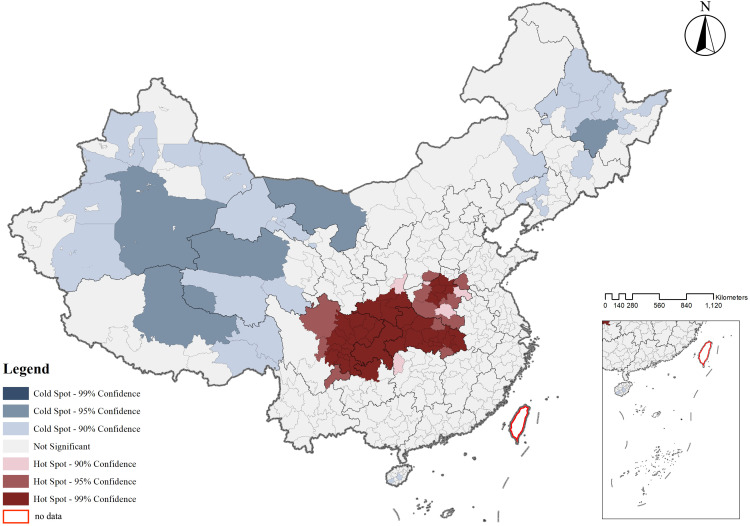
Map of cold hot spots of CATs.

The spatial differentiation pattern of CATs was significant, characterized by an interwoven distribution of hotspot and coldspot regions, with a spatial pattern of dispersed coldspots and concentrated hotspots. Based on the analysis of spatial data similarities or differences across various regions, further exploration of statistically significant clusters within prefecture-level units was conducted using the Local Association Index. Employing the Jenks natural breaks classification method, the spatial distribution of CATs was divided into coldspot and hotspot regions, each further classified into three distinct levels.The hotspot regions included 60 prefecture-level units such as Guang’an, Jingmen, and Yibin, accounting for 12.372% of the total national area. The coldspot regions encompassed 30 prefecture-level units such as Anshan, Aksu, and Yushu, covering 55.651% of the total national area (**Fig 7**).

**Fig 8:**
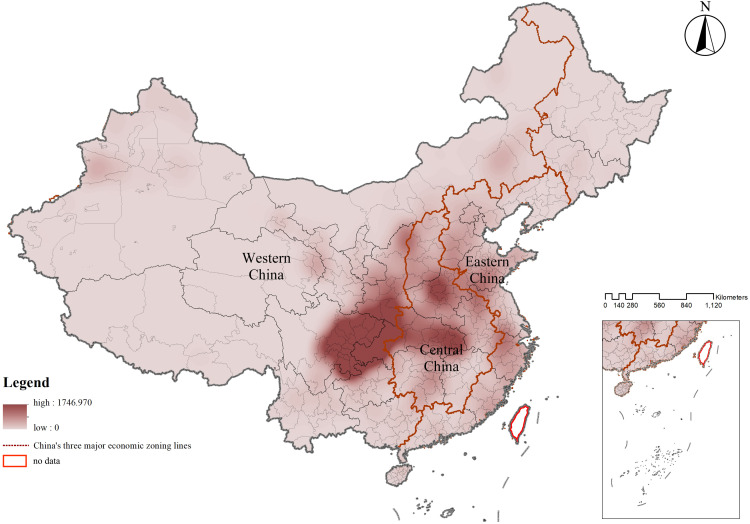
Kernel density of CATs in China’s three major economic zones.

In China’s three major economic lines, the number and density of CATs exhibited significant differences, with a decreasing trend in quantity from west to east, reflecting a “more in the west, less in the east; clustered in the west, scattered in the east” differentiation pattern. In terms of quantity, the western region accounted for the highest proportion of CATs, approximately 56.851%, followed by the central region at about 27.479%, and the eastern region with the lowest proportion at roughly 15.670%. Regarding density, the western region had the highest clustering degree of CATs, with a density of 0.080 units/km². The central and eastern regions had densities of approximately 0.015 units/km² and 0.013 units/km² (**Table 3** and **Fig 8**).

**Table 3:** Distribution of immovable cultural relics in China’s three major economic lines.

District	Density of CATs (units/km^2^)	Number of CATs	Percentage of the number of CATs
Eastern Region	0.013	14748	15.670%
Central Region	0.015	25862	27.479%
Western Region	0.080	53505	56.851%

**Fig 9:**
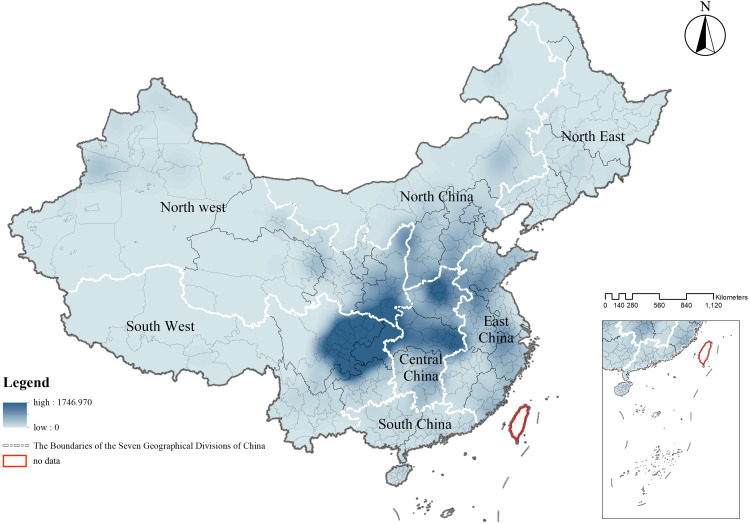
Kernel density of CATs distribution in seven geographical divisions of China.

Based on the analysis of The Seven Geographical Divisions of China, significant differences were observed in the number and density of CATs. The Southwest region had the highest proportion and density of CATs, while the Northeast region had the lowest proportion and density. In terms of distribution quantity, the proportions of CATs in Central China, East China, South China, North China, Northwest, Southwest, and Northeast regions accounted for 19.752%, 14.183%, 2.177%, 9.809%, 15.498%, 37.377%, and 1.204% of the total number of CATs nationwide, respectively. Among these, the Southwest region had the highest proportion, while the Northeast region had the lowest. In terms of distribution density, Central China and the Southwest region exhibited a relatively high clustering of CATs, with densities of 0.024 units/km² and 0.011 units/km², respectively. In contrast, South China, North China, Northwest, and Northeast regions displayed a more scattered distribution, with densities below 0.005 units/km². These findings demonstrate the regional differences in the distribution of CATs, characterized by an uneven distribution in both density and quantity (**Table 4** and **Fig 9**).

**Table 4:** Distribution of immovable cultural relics in seven geographical divisions of China.

District	Density of CATs (units/km^2^)	Number of CATs	Percentage of the number of CATs
Central China	0.024	18590	19.752%
East China	0.012	13348	14.183%
South China	0.004	2049	2.177%
North China	0.003	9232	9.809%
Northwest	0.003	14586	15.498%
Southwest	0.011	35177	37.377%
Northeast	0.001	1133	1.204%

**Fig 10:**
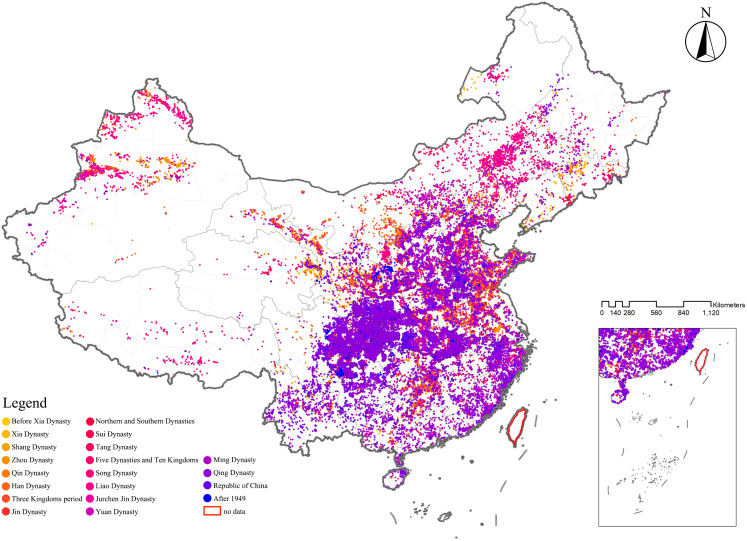
The spatial distribution of CATs across different historical periods.

**Fig 11:**
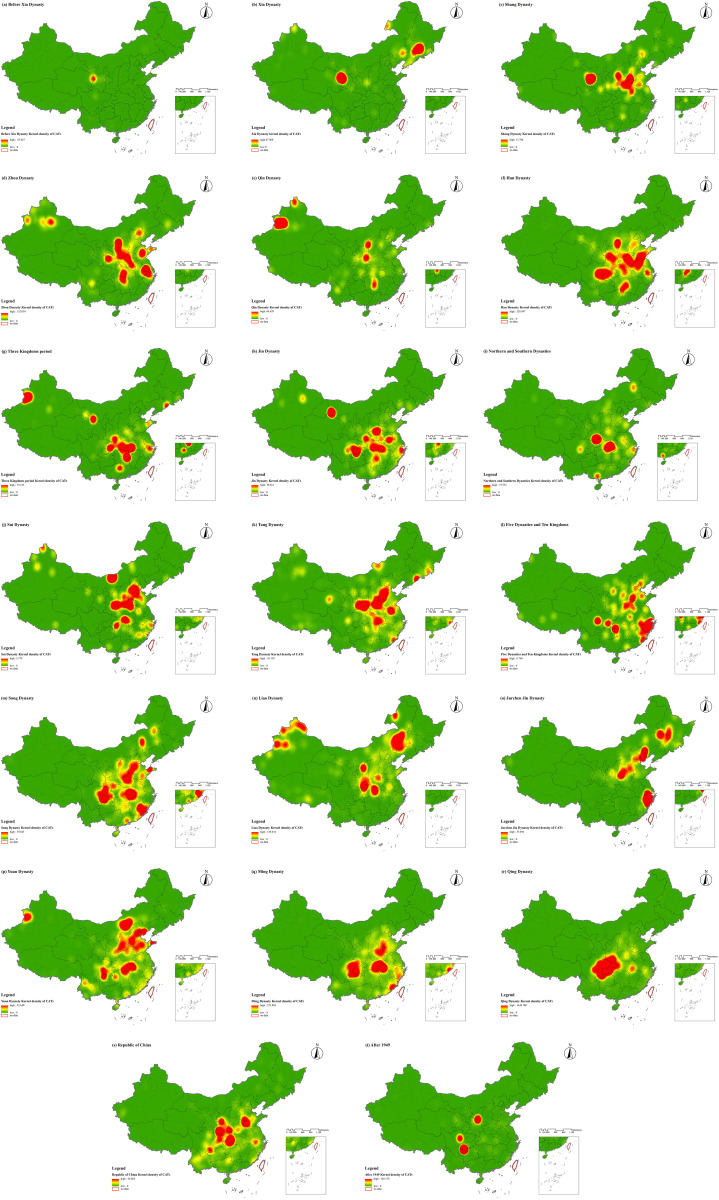
Kernel density analysis map of CATs across different historical periods.

**Spatial Distribution Characteristics of CATs in Different Historical Periods:** The spatial distribution of CATs exhibited distinct characteristics across different historical periods, closely associated with socioeconomic conditions and the shifts of political centers in each era. During the Qin Dynasty and earlier periods, CATs were characterized by multiple distribution centers and a relatively dispersed spatial pattern, with no significant clustering. At that time, various ethnic groups and communities coexisted within the territory of China, each with its unique cultural traditions and religious beliefs. These cultural differences were reflected in burial practices, as different groups chose burial locations based on their specific traditions and beliefs. Consequently, this led to a scattered distribution of tombs across regions. After the unification of the six states by the Qin Dynasty, from the Han Dynasty to the Sui and Tang Dynasties, tombs gradually became concentrated in the southern regions of the Yangtze River, particularly in Central and East China. During this period, China experienced cycles of centralization, division, and re-centralization. As history progressed, the distribution of tombs evolved correspondingly. Specifically, during the late Eastern Han Dynasty through the Wei, Jin, and Southern and Northern Dynasties, frequent warfare in the north caused large-scale population migrations to the relatively stable southern regions. These areas experienced economic development and became new political and economic centers, directly promoting the clustering of tombs in the south. During the Five Dynasties and Ten Kingdoms period, the fragmentation of China once again resulted in a multi-centered pattern of tomb distribution. During the Song Dynasty, which overlapped in time with the Liao and Jin Dynasties, the political center of the Song was located in the south, while the Liao, Jin, and Yuan Dynasties were centered in the north. This caused the distribution of tombs during this period to shift slightly toward the northeast. After the Ming Dynasty, the distribution of CATs increasingly concentrated in Central and Southwest China. During this time, as the national economy developed, the Central and Southwest regions rose as important economic areas. Economic prosperity in these regions attracted significant populations, providing the foundation for the construction of tombs. In summary, studying the spatial distribution characteristics of CATs in different historical periods is of great significance. Such research not only reveals the cultural, economic, and political conditions of ancient societies but also provides valuable insights into the lifestyles and social structures of past populations (**Fig 10** and **Fig 11**).

#### 3.1.3. Spatiotemporal evolution characteristics.

This study employed the standard deviational ellipse (SDE) analysis method within the ArcGIS platform to analyze the directional trends and distribution patterns of CATs across different historical periods. By utilizing SDE, the study aimed to explore the spatial shift trajectories and movement distances of the central points of tomb distributions over time. SDE is a spatial statistical method used to quantitatively describe the spatial distribution characteristics of geographic features, including centrality, directionality, and spatial morphology. This method generates an ellipse to represent the orientation and distribution characteristics of a dataset. The ellipse is defined by two key parameters: the major axis and the minor axis, which respectively indicate the primary direction of the data distribution and the degree of concentration. Through the application of SDE analysis to the distribution of CATs across various historical periods, it became possible to visually depict their spatial central positions, primary orientations, and morphological characteristics. The area of the SDE serves as an indicator of the spatial clustering characteristics of tombs during different periods. The centroid coordinates represent the geographic center of tomb distributions in a given period, while the standard distances, corresponding to the lengths of the major and minor axes, reflect the primary and secondary directions of spatial distribution. The flatness ratio, defined as the ratio of the minor axis to the major axis and ranging between 0 and 1, reveals the degree of directionality in the data. A higher flatness ratio indicates a stronger directional trend, with tombs being more concentrated along a specific direction. Conversely, a lower flatness ratio suggests a more evenly distributed dataset with weaker directionality. Additionally, the rotation angle of the ellipse, measured clockwise from true north (0°), indicates the primary directional trend of tomb distributions. This angle provides insight into the dominant spatial orientation of tombs in different historical periods (**Table 5**).

**Table 5:** The centroid coordinates, rotation angles, and flattening ratios of CATs across different historical periods.

Dynasty	Centroid Coordinates		Standard Distance		Flattening Ratio	Rotation Angle
	XCoord	YCoord	X StdDist	Y StdDist		
Before Xia Dynasty	106.369	36.192	5.045	9.629	0.48	74.025
Xia Dynasty	111.554	39.682	5.509	17.523	0.69	78.119
Shang Dynasty	110.227	35.861	4.356	9.683	0.55	88.863
Zhou Dynasty	111.053	35.237	14.568	5.695	0.61	106.904
Qin Dynasty	97.877	38.463	21.758	5.645	0.74	107.432
Han Dynasty	111.731	33.364	5.082	7.448	0.32	79.390
Three Kingdoms Period	107.171	33.680	19.329	5.718	0.70	108.250
Jin Dynasty	110.402	32.362	10.836	5.497	0.49	110.137
Southern and Northern Dynasties	112.486	32.441	5.751	7.003	0.18	53.310
Sui Dynasty	110.977	36.055	11.753	6.016	0.49	117.370
Tang Dynasty	112.208	34.685	7.169	11.466	0.37	88.248
Five Dynasties and Ten Kingdoms Period	113.958	31.365	9.861	5.982	0.39	99.285
Song Dynasty	113.115	32.2919	6.624935	8.452	0.22	39.220
Liao Dynasty	107.998	39.451	18.96663	8.528	0.55	93.166
Jurchen Jin Dynasty	118.672	36.359	7.621135	9.636	0.21	36.079
Yuan Dynasty	111.485	34.380	12.117157	8.402	0.31	94.807
Ming Dynasty	112.226	30.834	5.697372	7.278	0.22	79.914
Qing Dynasty	108.744	30.946	3.972855	6.518	0.39	72.938
Republic of China	110.489	31.578	5.594627	8.489	0.34	72.992
After 1949	106.565	30.412	4.723262	6.866	0.31	59.799

**Fig 12:**
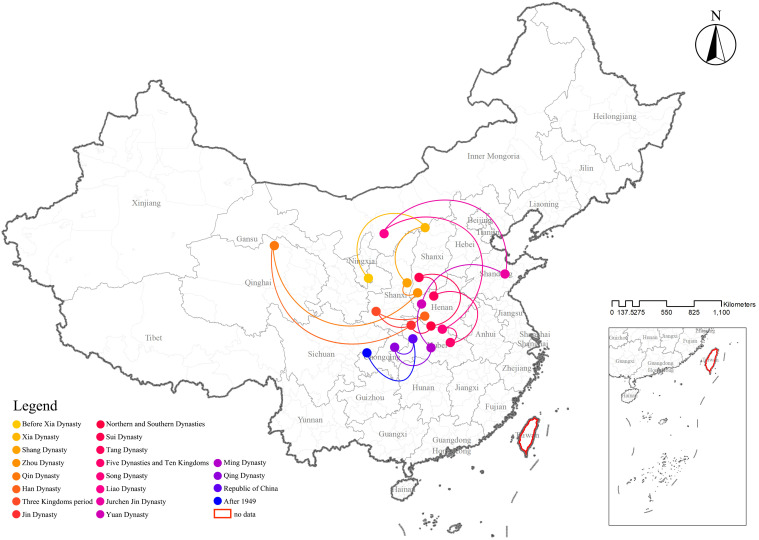
The centroid shift trajectory of CATs across different historical periods.

**Fig 13:**
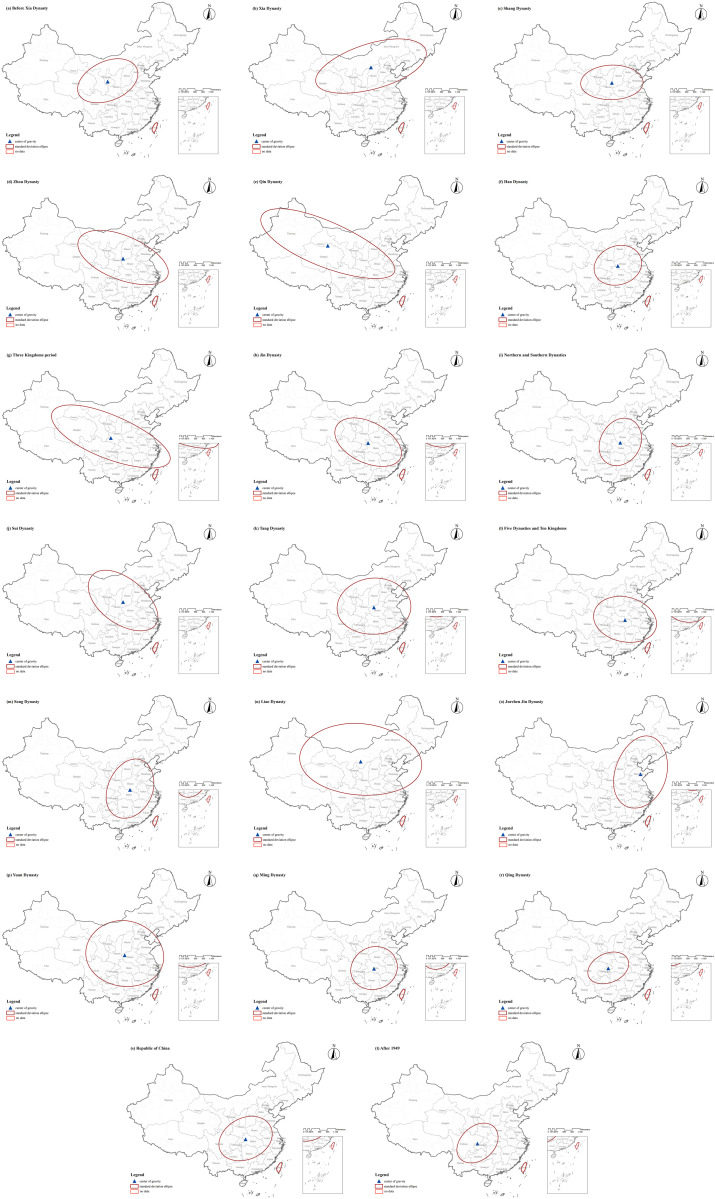
The directional distribution and centroid of CATs across different historical periods.

The spatial center of CATs distributions underwent dynamic shifts over time, influenced by changes in historical and political centers. Before the Yuan Dynasty, the distribution center alternated between the northern and southern regions, while after the Yuan Dynasty, it gradually shifted southwestward (**Fig 12**). Before the Qin Dynasty, the tomb distribution center was concentrated in northern China. During the Han Dynasty, it began to shift southward. In the early Han period, efforts to consolidate governance and defend against incursions from the Xiongnu in the north led to measures such as migration to border regions and the establishment of military garrisons. At the same time, factors such as war and natural disasters prompted some Han populations to migrate southward. These migrating Han communities brought the burial culture and customs of the Central Plains to the south, contributing to the southward shift of the tomb distribution center. From the Three Kingdoms to the Five Dynasties, China experienced a series of cycles of unification and division. After the fall of the Eastern Han, the Three Kingdoms emerged, characterized by fragmented regional power. Although the Western Jin briefly unified the country, it quickly fell into another period of division, with the north engulfed in prolonged warfare and instability, leading to the chaotic era of the Five Barbarians and Sixteen Kingdoms. During the Southern and Northern Dynasties, the south witnessed successive dynasties—Song, Qi, Liang, and Chen—while the north saw the rise and fall of regimes such as the Northern Wei, Eastern Wei, Western Wei, Northern Qi, and Northern Zhou. After the reunification of China under the Sui and Tang Dynasties, the country again fragmented during the late Tang period, entering the Five Dynasties and Ten Kingdoms era. The frequent shifts in political centers and the impact of warfare caused the tomb distribution center to alternate between northern and southern regions during this period, remaining concentrated in the Shaanxi and Hubei regions. During the Song, Liao, Jin, and Yuan periods, the tomb distribution center stabilized in the south. Despite the Liao and Jin Dynasties primarily governing northern China, their political and cultural influence on tomb distribution kept the spatial center in the north. The Yuan Dynasty, although ruled by a northern minority group, unified both northern and southern China. Due to the economic prominence of the south during this period, the tomb distribution center remained in southern regions. After the Ming Dynasty, the tomb distribution center gradually stabilized, concentrating in the southwest and central regions of China (**Fig 13**).

### 3.2. Influencing factors

#### 3.2.1. Selection of influencing factors.

The spatiotemporal distribution patterns of CATs were deeply influenced by the interplay of multiple factors, including the shaping effects of natural geographic conditions and the driving forces of sociocultural factors [29]. To further analyze this complex distribution pattern, this study selected indicators from eight dimensions: socioeconomic factors, historical and cultural influences, river systems, climatic conditions, topography and geomorphology, transportation networks, vegetation cover, and soil types. The aim was to comprehensively uncover the multifaceted causes and the interaction mechanisms underlying the distribution of CATs (**Table 6**).

**Table 6:** Selection of influencing factors.

Group	Dimension	Influencing Factors
Socio-cultural	Socioeconomic	Total Population
		Total GDP
	Historical-cultural	The Number of Regional Historical Figures
		Density of Traditional Village
		Density of Administrative Villages
Natural geographic	River Systems	Water Network Density
	Climatic Conditions	Annual Average Temperature
		Annual Average Precipitation
		Total Runoff
		Average Value of Runoff
	Topography and Landforms	Average Slope
		Average Elevation
	Road Networks	Network Density
	Vegetation Coverage	Vegetation Coverage
	Soil types	The Density of Southern Paddy Soils (DL01)
		Freshly Irrigated Paddy Soils (DL02)
		Northern Paddy Soils (DL03)
		Northern Paddy Yellow Clay Soils (DL04)
		Yellowish Paddy Soils (DL05)
		Loessial Clay Soils (DL06)
		Lou Soils (DL07)
		Black Loessial Clay Soils (DL08)
		Loamy Soils (DL09)

The socioeconomic dimension within sociocultural factors encompasses key indicators such as total population and GDP. As CATs are direct products of human historical activities, their existence, distribution, and subsequent inheritance and preservation are deeply rooted in human socioeconomic activities. Consequently, variations in population size and economic output profoundly influenced the distribution patterns of CATs. In the dimension of historical and cultural factors, the number of regional historical figures serves as an important reference indicator. Historical figures act as catalysts for cultural exchange and participants in historical events, often making their areas of activity focal points for cultural and historical convergence. This status not only directly affected the distribution and quantity of CATs but also profoundly reflected the historical and cultural accumulation and the degree of cultural prosperity in various regions.

Natural geographic are also a critical category of influences. Within the river system dimension, a key factor is water network density, which reflects the degree of development of the regional river network system. During the evolution of ancient civilizations, water networks played a pivotal role, not only providing essential water resources for survival but also serving as transportation routes. Regions with dense water networks, due to their abundant natural resources and convenient water transport conditions, often became preferred locations for ancient settlements, which in turn might have given rise to more CATs. The climate dimension incorporates key indicators such as total runoff, annual average temperature, and annual average precipitation. These factors directly influenced the preservation state of CATs and also impacted the complexity and feasibility of archaeological excavation work. The topography and geomorphology dimension includes average slope and average elevation, both of which played a decisive role in determining the distribution patterns and long-term preservation of CATs. In the transportation dimension, road network density stands out as an essential element. A well-developed transportation network undoubtedly facilitated the protective excavation of CATs, promoting the research and dissemination of cultural heritage. However, it is equally important to be mindful of its potential negative impact, as excessive exposure brought about by extensive transportation networks may increase the risk of damage to CATs. The vegetation dimension highlights the significance of vegetation coverage. Dense vegetation can effectively conceal CATs to a certain extent, providing a degree of protection. However, it can also pose a challenge in the process of archaeological discovery. Finally, soil characteristics have a significant impact on the stability, preservation state, and excavation efforts related to CATs. This study selected nine types of soil, including the density of southern paddy soils (DL01), freshly irrigated paddy soils (DL02), northern paddy soils (DL03), northern paddy yellow clay soils (DL04), yellowish paddy soils (DL05), loessial clay soils (DL06), Lou soils (DL07), black loessial clay soils (DL08), and loamy soils (DL09). These soil types were crucial for understanding the relationship between soil properties and the distribution and preservation of CATs.

#### 3.2.2. Determination of influencing factors.

Ridge regression is a biased estimation method specifically designed to handle collinear data analysis. Its core concept involves adding a regularization term to the loss function of traditional ordinary least squares regression. This regularization term imposes a constraint on the magnitude of regression coefficients, aiming to reduce model complexity, prevent overfitting, and enhance the stability of coefficient estimates. Specifically, ridge regression adjusts the estimation of regression coefficients, making the model more robust when dealing with multi-collinearity issues. The use of the Variance Inflation Factor (VIF) to measure multi-collinearity in multiple linear regression models is an effective approach. A higher VIF value indicates a greater likelihood of collinearity among independent variables, which can lead to unstable coefficient estimates and increased model error. If any VIF value exceeds 10, it suggests the presence of a collinearity problem in the corresponding variable. The table below (**Table 7**) presents the results of collinearity analysis using VIF values, demonstrating the existence of multi-collinearity issues.

**Table 7:** Collinearity diagnostic table of influencing factors for the distribution of CATs.

Projects	VIF value	Tolerance
Number of CATs	1.601	0.625
DL01	12.771	0.078
DL02	6.093	0.164
DL03	3.251	0.308
DL04	2.509	0.399
DL05	3.041	0.329
DL06	1.602	0.624
DL07	1.722	0.581
DL08	2.775	0.360
DL09	3.562	0.281
Vegetation Coverage	3.656	0.273
Total GDP	4.400	0.227
Total Population	4.571	0.219
The Number of Regional Historical Figures	1.896	0.527
Annual Average Precipitation	2.911	0.344
Annual Average Temperature	4.151	0.241
Average Slope	3.404	0.294
Average Elevation	4.608	0.217
Water Network Density	1.351	0.740
Network Density	1.295	0.772
Density of Traditional Village	1.480	0.676
Density of Administrative Villages	1.267	0.789
Average Value of Runoff	1.457	0.686
Total Runoff	1.456	0.687

Note: Cells with a red background indicate cases where VIF > 10 or null values are present.

**Fig 14:**
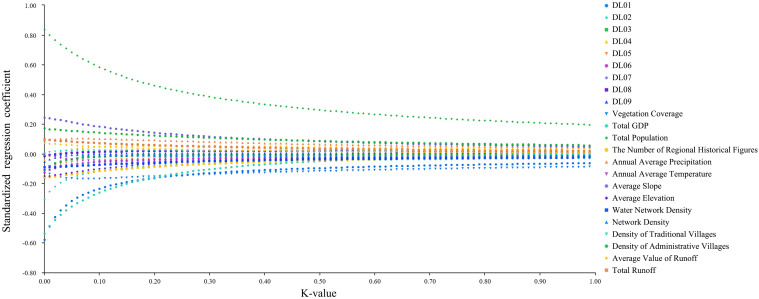
Ridge trace plot of influencing factors for the distribution of CATs.

This study first utilized ridge regression analysis to determine the relationship between the number of CATs and various influencing factors, as well as to verify the rationality of these factors. In this analysis, the number of CATs was set as the dependent variable, while the influencing factors were treated as independent variables. A ridge trace plot was then obtained (**Fig 14**). The ridge regression ANOVA test (also known as the F-test) was conducted with (K) set to 0.010. The results showed a (P)-value of 0.001, which is less than 0.050, indicating that the model was statistically significant (**Table 8**). This confirmed the presence of a regression relationship between the independent and dependent variables. Among the factors, total population, DL01, and total GDP passed the 5% significance level test, suggesting that these three factors had a substantial influence on the spatial distribution of CATs and showed strong explanatory power (**Table 9**).

**Table 8:** Ridge regression ANOVA test.

Projects	Sum of Squares	*df*	Mean Square	*F*	*p-*value
Regression	111219738.956	23	4835640.824	2.625	0.001
Residual	186040933.652	101	1841989.442		

**Table 9:** Ridge regression analysis results.

Projects	Non-standardized coefficient		Standardized coefficient	T	p	VIF value
	B	Standard Error	Beta			
Total GDP	−0.073	0.022	−0.488	−3.365	0.001**	3.390
Total Population	2.411	0.418	0.799	5.771	0.000**	3.095
The Number of Regional Historical Figures	−0.174	0.115	−0.157	−1.507	0.135	1.745
Density of Traditional Village	0.357	3.172	0.010	0.113	0.911	1.383
Density of Administrative Villages	0.166	0.086	0.165	1.931	0.056	1.183
Annual Average Precipitation	0.295	0.399	0.095	0.738	0.462	2.668
Annual Average Temperature	−4.171	41.284	−0.015	−0.101	0.920	3.660
Average Slope	54.171	30.478	0.238	1.777	0.079	2.892
Average Elevation	−0.229	0.239	−0.149	−0.958	0.340	3.900
Water Network Density	−982.241	978.121	−0.090	−1.004	0.318	1.284
Network Density	−17.266	177.097	−0.009	−0.097	0.923	1.244
DL01	−1877.861	867.841	−0.489	−2.164	0.033*	8.258
DL02	−1348.359	859.031	−0.256	−1.570	0.120	4.295
DL03	−694.786	1059.268	−0.083	−0.656	0.513	2.555
DL04	−140.531	1122.814	−0.014	−0.125	0.901	1.969
DL05	−1018.018	1382.648	−0.092	−0.736	0.463	2.495
DL06	−2188.044	1873.022	−0.109	−1.168	0.245	1.405
DL07	−3988.357	4175.412	−0.094	−0.955	0.342	1.560
DL08	−713.858	9482.392	−0.009	−0.075	0.940	2.535
DL09	−460.799	1733.807	−0.036	−0.266	0.791	3.015
Vegetation Coverage	−1375.923	1360.734	−0.135	−1.011	0.314	2.896
Average Value of Runoff	0.239	0.325	0.068	0.734	0.465	1.386
Total Runoff	0.010	0.010	0.092	0.995	0.322	1.378
R^2^	0.374					
F	F (23,101)=2.625,p = 0.001					

Note: Dependent variable = Number of CATs

* p < 0.050 ** p < 0.01

#### 3.2.3. Validation of influencing factors.

Geographically Weighted Regression (GWR), as a spatial analysis technique, offers significant advantages over traditional global regression methods. The GWR model accounts for the local effects of spatial objects by constructing independent regression equations for each geographic location, thereby enhancing the accuracy and explanatory power of the model. Therefore, this study incorporates the GWR model based on ridge regression to further improve the scientific rigor and precision of the analysis.

First, a global spatial autocorrelation analysis was conducted on the three influencing factors (total population, total GDP, and DL01) that passed the significance test at the 5% level within the study area. This analysis provides a comprehensive measure of the spatial data across the entire study region, reflecting whether the data exhibit a clustering or dispersion trend and the strength and significance of such trends. The results indicated that the Moran’s I indices and z-values for all factors were greater than 0, and the p-values were less than 0.050 (p < 0.05, statistically significant), suggesting that the distribution of ancient Chinese tombs and the three influencing factors demonstrated significant global spatial autocorrelation. In this study, the VIF values for all three influencing factors were less than 5, indicating the absence of or low levels of multi-collinearity (**Table 10**).

**Table 10:** Significance test of global spatial autocorrelation and multi-collinearity diagnostics table for CATs.

Projects	Moran’sI	z	p	VIF value
Number of CATs	0.079	10.151	0.000	–
DL01	0.040	4.314	0.000	1.056
Total GDP	0.002	0.488	0.026	1.003
Total Population	0.019	2.314	0.020	1.057

Subsequently, a geographically weighted regression (GWR) analysis was conducted on the three influencing factors that passed the significance test at the 5% level. The number of CATs within the study area was used as the dependent variable, while the three influencing factors were used as independent variables to construct the GWR model. The coefficient of determination R^2^ serves as a measure of goodness of fit, reflecting the proportion of variation in the dependent variable explained by the model. The adjusted R^2^ is considered a more reliable measure of goodness of fit. In this study, the R^2^ and adjusted R^2^ values of the GWR model were 0.748 and 0.672, respectively, both exceeding 0.6. This indicates that the GWR model has strong explanatory power, confirming the significant correlation between the number of CATs and the three influencing factors (**Table 11**).

**Table 11:** Determination coefficients of the geographically weighted regression model.

R^2^	Adjusted R^2^	AICc
0.748	0.672	5925.097

**Fig 15:**
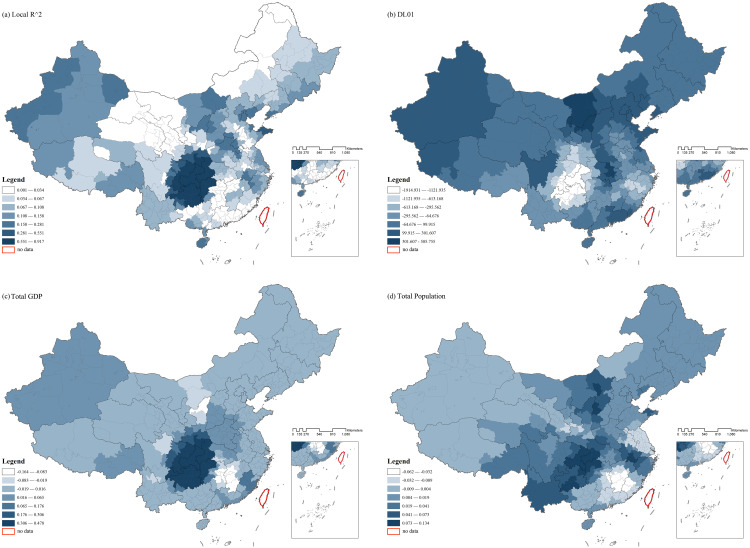
Geographically weighted regression model.

Regression coefficients represent the degree of correlation and the direction (positive or negative) of the relationship between influencing factors and the dependent variable (**Table 12** and **Fig 15**).

**Table 12:** Statistical results of geographically weighted regression coefficients.

Projects	Average value	Maximum value	Minimum value	Standard Error
Local R^2^	0.144	0.917	0.001	0.192
DL01	−94.158	585.755	−1914.931	376.161
Total GDP	0.045	16.759	−0.164	0.119
Total Population	0.015	0.134	−0.062	0.028

The regression coefficient for total population ranged from −0.060 to 0.130, with an average value of 0.015. The majority of regions exhibited positive regression coefficients, indicating that total population was generally positively correlated with the number of CATs. Regions with high regression coefficients for total population were located in the urban clusters of the middle and lower Yangtze River, Chengdu-Chongqing, Hohhot-Baotou-Ordos-Yulin (HBY), and the Central Plains. Conversely, regions with low regression coefficients were primarily found in western China and southeastern coastal cities.

The regression coefficient for total GDP ranged from −0.160 to 16.760, with an average value of 0.040. Most regions had positive regression coefficients, suggesting a general positive correlation between total GDP and the number of CATs. High-value regions for GDP regression coefficients were primarily concentrated in the Chengdu-Chongqing urban cluster, while low-value regions were observed in Hunan, Hubei, northern Shanxi, and central Inner Mongolia.

The regression coefficient for DL01 ranged from −1914–585, with an average value of 0.015. However, most regions exhibited negative regression coefficients, indicating that DL01 was generally negatively correlated with the number of CATs. Regions with high DL01 regression coefficients were located in northern China and the Central Plains urban cluster, while low-value regions were concentrated in the Chengdu-Chongqing urban cluster.

**Fig 16:**
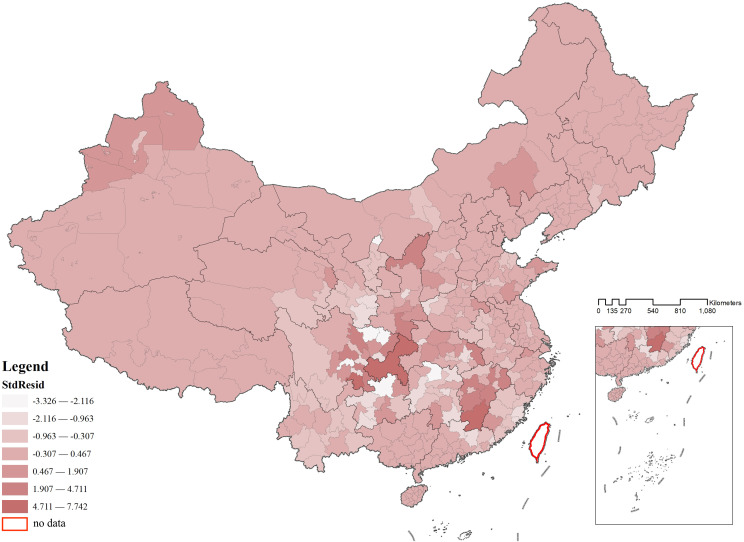
Spatial distribution of standardized residuals for the distribution of CATs.

**Fig 17:**
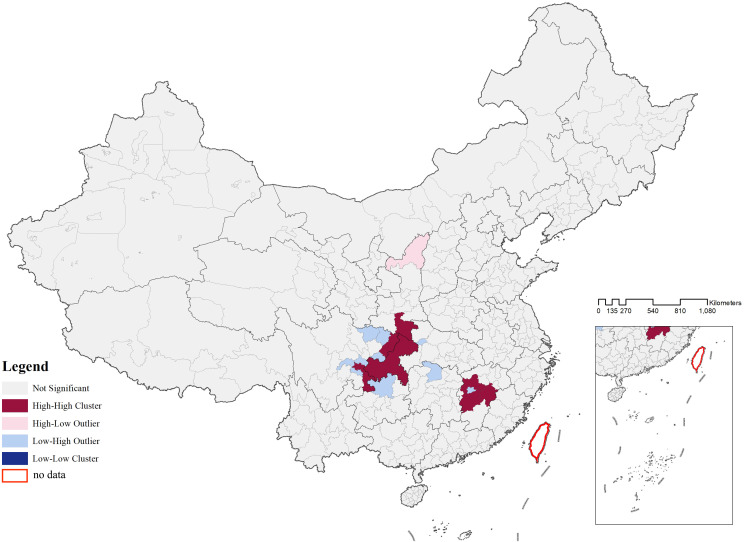
Standardized residual clusters and spatial outliers of CATs distribution.

Using the spatial distribution of standardized residuals, the regression performance of the GWR model was further interpreted. The results indicated that residuals were not significant in most areas and were randomly dispersed (**Fig 16**). This suggested that the GWR model had addressed the majority of spatial heterogeneity issues. However, a small number of areas still exhibited clustering and spatial outliers, indicating the presence of spatial heterogeneity in these regions or the necessity of incorporating additional influencing factors as variables into the model (**Fig 17**).

## 4. Discussion

### 4.1. The fluctuating patterns of the number of CATs

The fluctuation patterns and influencing factors of the number of ancient tombs in China across different historical periods were multifaceted. Previous related studies indicated that factors such as sociopolitical and economic contexts, as well as architectural technologies, intertwined and influenced one another, collectively shaping this unique form of cultural heritage represented by ancient tombs. A deeper exploration of the underlying regularities of this phenomenon can not only reveal the spatial distribution of CATs during various historical stages but also reflect the regional distribution and intensity of human activity during each period.

Firstly, socio-political factors had the greatest impact on the number of CATs. During periods of political stability, effective governance, and a clear political environment, the number of preserved cultural relics and monuments was relatively high [23]. Several periods in Chinese history saw rapid increases in the number of tombs, including the Qin-Han, Sui-Tang, and Yuan-Ming-Qing dynasties. During these times, the ruling class had largely unified the country, resulting in political stability and economic prosperity, thus creating favorable conditions for large-scale tomb construction. In contrast, during periods of division, such as the Three Kingdoms, Jin-Southern and Northern Dynasties, and Five Dynasties and Ten Kingdoms, frequent warfare and social instability often restricted tomb construction, leading to a relative decrease in their number.

Secondly, periods of economic prosperity also contributed to an increase in tomb construction. As people’s living standards improved, so did their attention to the afterlife, further promoting the development of tombs. In the mid-Western Han period, especially during Emperor Wu’s reign, after a long period of “nurturing and recovery” policies, the economy rapidly recovered and developed. During the Tang dynasty, the economy reached unprecedented heights, and the unimpeded Silk Road facilitated trade. At this time, all social classes accumulated considerable wealth, which further promoted tomb construction. The Song dynasty marked a period of high economic and cultural development in Chinese history, with Gongyi in Henan, as one of the economic centers, witnessing prosperous tomb construction. These periods of economic growth saw a marked increase in the number of CATs. Ancient tombs were better protected during periods of economic prosperity, resulting in a notable increase in their numbers during these times of significant economic development.Additionally, advancements in construction techniques also played a significant role in promoting tomb construction. For instance, during the Qin-Han period, there was a sharp increase in the number of tombs, partly due to the widespread application of rammed earth technology, which facilitated the construction of high platforms and large tombs. These tombs were not only grand in scale but also complex in structure, reflecting the advanced architectural techniques of the time. By the Tang dynasty, brick and stone construction techniques had further developed and became the mainstream for tomb construction. The use of brick and stone was not only sturdy and durable but also had excellent moisture and pest-resistant properties, ensuring the long-term preservation of tombs. The Ming-Qing period was a culmination of ancient Chinese architectural techniques, with both brick-and-stone and wooden structures reaching unprecedented heights, contributing to a significant increase in the number of tombs during this time [30].

### 4.2. Geographic differentiation of CATs

The spatial differentiation patterns and characteristics of CATs are evident. Studies have shown that high-density areas of ancient tombs are concentrated in the Chengdu-Chongqing urban cluster, the Guanzhong Plain urban cluster, and the Central Plains urban cluster. For example, research on the spatiotemporal distribution and causes of China’s architectural heritage has explored the distribution patterns of cultural heritage, although the objects of study were not specifically ancient tombs. These studies similarly identified Shanxi, Shaanxi, Henan, and the middle-lower Yangtze River Plain as regions with a high concentration of cultural heritage [31]. In this study, the Chengdu-Chongqing urban cluster emerged as the highest-density core area, while other high-density areas of CATs were distributed around the core points of northern Shaanxi and Shanxi, central Henan, and eastern Hubei. Such regional differentiation patterns are closely related to the natural geographical environment and historical cultural context.

Firstly, in terms of natural geographical conditions, both the Chengdu-Chongqing and Central Plains urban clusters have historically possessed favorable geological conditions, providing a natural foundation for the preservation of CATs. The terrain of the Chengdu-Chongqing urban cluster is dominated by hills and basins, which are relatively flat and characterized by fertile soil, facilitating ancient human settlements and agricultural production. The stable geological conditions also contributed to the preservation of tombs. Similarly, the Central Plains urban cluster features flat terrain and abundant water resources, offering ideal natural conditions for the development of ancient civilizations. This, in turn, attracted large populations, resulting in the formation of numerous ancient settlements and tomb clusters. In terms of climate, these high-density regions are predominantly located in temperate monsoon climate zones, which feature distinct seasons and moderate precipitation. Additionally, the relatively humid climate conditions in these areas were conducive to the preservation of coffins and burial artifacts within the tombs.

Secondly, CATs are products of human activity during historical periods, and their emergence and distribution were influenced by historical and cultural factors. Regions with dense distributions of CATs often possess a profound historical and cultural background [32]. The Chengdu-Chongqing area, for instance, has long been one of the cradles of Bashu culture. The ancient Bashu region experienced economic prosperity and cultural flourishing, giving rise to a unique tomb culture [33]. The Central Plains urban cluster, encompassing the middle and lower reaches of the Yangtze and Yellow Rivers, is another cradle of ancient Chinese civilization, hosting cultures such as the Yangshao and Longshan in the Yellow River basin and the Hemudu and Liangzhu cultures in the Yangtze River basin [34,35]. The development of these ancient civilizations provided a crucial historical context for the formation and evolution of tomb culture. Historically, these high-density regions were also political, economic, and cultural centers. For example, northern Shaanxi and Shanxi served as important political centers during the Qin-Han and Sui-Tang dynasties; central Henan was one of the birthplaces of Central Plains culture; and eastern Hubei was the core area of Chu culture. These regions, characterized by political stability, economic prosperity, and large populations, witnessed extensive tomb construction.

### 4.3. Spatial and temporal evolutionary patterns and characteristics of CATs

In previous studies, the spatiotemporal evolutionary patterns of different types of cultural heritage varied significantly. In this study, the centers of ancient tombs in China during different historical periods were mainly concentrated in the central region and repeatedly shifted within the present provinces of Shaanxi, Shanxi, Henan, Hubei, and Chongqing as history progressed. This phenomenon was related to factors such as the ruling classes, the stability of political regimes, and the transformations of authority in different historical periods. In the Shang and Zhou periods, the distribution of CATs was concentrated in Shanxi and Shaanxi. Shanxi was one of the major ruling areas during the Shang dynasty, while in the Zhou dynasty, the state of Jin rose to prominence in Shanxi. Jin’s ruler, Duke Wen, was one of the Five Hegemons during the Spring and Autumn period. Furthermore, the political center of the Western Zhou dynasty was in Haojing (present-day Xi’an, Shaanxi), which served as a political hub. This long-standing political centrality made Shanxi a significant area for the activities of nobles and royals, leading to the concentration of their tombs in this region. From the Han dynasty to the Five Dynasties period (spanning the Sui and Tang dynasties), the focus of CATs alternated between Henan, Hubei, Shaanxi, and Shanxi. During this time, China’s political centers underwent several shifts. For instance, both the Han and Tang dynasties established their capitals in Chang’an (present-day Xi’an, Shaanxi), making Shaanxi a political and cultural center. However, during the Sui and Tang periods, the political center gradually shifted to Luoyang (present-day Henan), further contributing to the development of CATs in Henan [36]. Hubei was the focal point of contention during the Three Kingdoms period. Later, it served as a significant ruling area for the Southern Dynasties, resulting in a substantial number of CATs in this region. The Song, Liao, and Jurchen Jin dynasty coexisted during roughly the same historical period, but their centers of rule differed, leading to variations in the focal regions of tomb distribution. During the Song dynasty, which unified southern China at the time, the focus of tombs was located near the border between Henan and Hubei. In the Jurchen Jin dynasty, efforts to consolidate control over the Central Plains involved repeated military campaigns and population relocations. Many of the migrating nobles and officials settled in Shandong, leading to a concentration of tombs in this area and making Shandong the focal point during the Jin period. In contrast, the Liao dynasty, established by the Khitan, governed northern regions, including present-day Inner Mongolia, Liaoning, and Jilin. Consequently, the Liao dynasty’s tomb distribution was concentrated in southern Inner Mongolia. During the Yuan dynasty and subsequent periods, as the political landscape was once again unified, the focus of CATs distribution gradually stabilized in the Hubei and Chengdu-Chongqing regions.

### 4.4. Factors affecting the distribution of CATs

Overall, the distribution of CATs was influenced by both cultural and natural factors, with the dominant influencing factors in this study being DL01, total population, and total GDP.

The number of CATs was negatively correlated with DL01. In existing studies on the spatiotemporal distribution of cultural relics in China, natural geographic factors such as precipitation, temperature, and slope are frequently selected, whereas the influence of soil types on the spatial distribution of cultural relics has been rarely analyzed. However, soil type is a crucial indicator of a region’s natural geological conditions, particularly because ancient tombs are often underground archaeological sites preserved for long periods in soil-covered environments [2]. Therefore, the soil environment significantly impacts CATs. Since rice cultivation was the primary agricultural activity in ancient southern China, lands with high paddy soil density were extensively used for rice production. As a result, regions with a relatively small number of CATs often correspond to areas with higher paddy soil density.

The number of CATs was positively correlated with the total population. The findings of this study align with previous research [32,37], which suggests that population density contributes to a relatively concentrated distribution of cultural relics, and that population has a positive correlation with the distribution of cultural heritage. From a temporal perspective, periods of frequent dynastic transitions and wars in Chinese history were often accompanied by significant population declines and migrations. However, during post-war recovery and periods of societal stability, population numbers typically rebounded and grew rapidly, which was also reflected in the increasing number of deceased individuals requiring burial. From a geographic perspective, areas such as the Central Plains urban cluster and the Chengdu-Chongqing urban cluster have historically been densely populated. These population-dense regions naturally saw a higher concentration of CATs, whereas sparsely populated areas correspondingly had fewer CATs.

The total GDP was positively correlated with the distribution of CATs. This finding is consistent with other related studies [23,38], which suggest that the level of economic development had a decisive impact on people’s production and living activities in historical periods. Periods with higher economic development levels tended to preserve a greater number of cultural heritage sites. In both modern and ancient societies, regions with higher economic levels often had higher population densities, which, in turn, resulted in the construction of more tombs. For researchers studying the preservation of CATs, conducting excavation work in economically developed areas may be more efficient, as tombs in these regions are more likely to be successfully discovered and preserved. This offers valuable perspectives and insights for researchers in formulating preservation strategies, guiding excavation efforts, and exploring the relationship between CATs and historical economic activities.

### 4.5. Research limitations

Although this study provides a certain basis for explaining the distribution of CATs using modern natural and cultural data, it still has limitations. First, it is difficult to obtain data from ancient times. Since CATs are mostly historical relics, their formation and distribution are often closely related to complex factors such as the natural environment and socio-economic conditions of the time. However, such ancient data are often hard to access due to the long time elapsed, lack of records, or poor preservation. While modern natural and cultural data, such as climatic characteristics, road network density, total population, and economic activities, can partially reflect the current natural environment and social conditions, they are challenging to directly correspond to specific ancient contexts.

In addition, the acquisition and processing of modern natural and cultural data have their own limitations. Although modern technological tools like Geographic Information Systems (GIS) provide abundant data sources for historical research, these data are subject to various influencing factors during their acquisition and processing, such as data accuracy, data completeness, and data interpretation. These factors may lead to inaccuracies or incompleteness in the data, thereby affecting the explanation of CATs.

## 5. Conclusions

Based on the above, this study utilized ancient tomb data from the Third National Cultural Relics Census of China to construct a spatial database of Chinese ancient tombs. Using qualitative and quantitative methods supported by ArcGIS and SPSS platforms, it examined the spatiotemporal distribution patterns and influencing factors of ancient tombs across China. The main conclusions of this research are as follows: ① Influenced by historical developments, economic conditions, and the degree of tomb preservation, the number of ancient tombs varied significantly across historical periods. Among them, the Qing dynasty had the highest number of tombs, accounting for 47.012% of the national total, while the Sui dynasty had the fewest, representing only 0.134%. ② The spatial distribution of ancient tombs in China exhibited significant regional differentiation, forming three prominent clusters around the Central Plains urban agglomeration in central China, the Chengdu-Chongqing urban agglomeration in the southwest, and the Guanzhong Plain urban agglomeration. A distinct core–periphery distribution pattern was observed. The high-value areas of kernel density also shifted over time across historical periods, stabilizing in the Chengdu-Chongqing region after the Ming dynasty, reflecting historical changes in human activity and settlement patterns. ③ The spatiotemporal distribution centers of ancient tombs during different historical periods were concentrated in central China. The center of distribution repeatedly shifted within present-day Shaanxi, Shanxi, Henan, Hubei, and Chongqing, driven by factors such as changes in ruling powers and political stability across different dynasties. ④ The distribution of ancient tombs in China was influenced by both human and natural factors. There was a negative correlation between the density of paddy soils in southern China (DL1) and the number of ancient tombs, while GDP and total population showed a significant positive correlation with the spatial distribution of ancient tombs.

This study conducted a systematic digital survey and analysis of the spatiotemporal distribution characteristics and influencing factors of ancient tombs in China, revealing their evolution patterns and influencing factors, thus laying an important theoretical foundation for building a scientific and precise protection system. In the future, the protection of ancient tombs in China urgently needs to transition from passive rescue to proactive preventive protection: First, establish a dynamic monitoring system based on spatiotemporal distribution patterns. By utilizing GIS spatial analysis technology, combined with historical environmental data and real-time monitoring information, key monitoring and early warning should be implemented in high-density and high-risk areas. Second, deepen the development of protection strategies driven by big data. Integrating information from archaeological excavations, historical records, environmental geology, and socio-economic factors, a spatiotemporal distribution prediction model and a risk assessment model for ancient tombs should be constructed. By analyzing the correlation between historical and current data, potential threat areas can be anticipated, protection resource allocation can be optimized, and differentiated protection strategies can be formulated for different zones. Third, build an open and shared data platform. Promote the digitalization, standardization, and platform-based sharing of basic information, monitoring data, and research findings of ancient tombs, providing solid data support for interdisciplinary research, public participation, and long-term protection decision-making.

In summary, the deep integration of this study’s understanding of spatiotemporal distribution patterns with advanced big data analysis methods represented a key pathway to enhancing the scientific, forward-looking, and efficient nature of ancient tomb protection in China. It provided a solid scientific foundation for informed decision-making, ensuring the sustainable preservation of ancient tombs and broader cultural heritage across the country.

## References

[1] ICOMOS C. The principles for the conservation of heritage sites in China. Beijing: Cultural Relics Press. 2015.

[2] Wang Z. The application of virtual reality technology in the protection and display of mausoleums. Fashion Color. 2022;5:25–7.

[3] Lin B. Research on the protection path and countermeasures of historical and cultural heritage ——— taking the ancient tombs in Hefei as an example. Journal of Jining University. 2024;45(3):34–9.

[4] Guo S. Suggestions on the Protection of Ancient Burial Tourism Resources in Shanxi Province. Modern Economic Information. 2014;(2):321.

[5] Wang Y, Jiang Y. The practical dilemma and improvement path of punishing the organized crimes on cultural relics———taking the perspective of crimes of excavating and robbing ancient cultural sites and tombs. Journal of Shanxi Police College. 2024;32(4):58–64.

[6] Chen W, Qi Q, Guo Z, Zhang S, Wu H, Liu P. Spatial and temporal distribution characteristics of single earthen heritage sites in Gansu Province. Journal of Lanzhou University (Natural Sciences). 2024;60(2):205–13. doi: 10.13885/j.issn.0455-2059.2024.02.009

[7] Han Y, Jia L, Zhang C, Lin C, Zhao P. Spatial distribution characteristics of ancient architecture cultural tourism resources in Shanxi. Journal of Arid Land Resources and Environment. 2021;35(1):196–202. doi: 10.13448/j.cnki.jalre.2021.029

[8] Gong Y, Wang L, Wang J. Spatial and temporal distribution characteristics of Chinese cave temple based on ArcGIS geographic information system analysis. Cultural Relics in Southern China. 2021;2021(4):256–63.

[9] Zhen S, Zeng L. Research on the conservation and exhibition utilization of cultural heritage of non-popular ancient tombs--the case of No.1 tomb of Emperor Qin Gong. Identification and appreciation to cultural relics. 2024;18:27–31. doi: 10.20005/j.cnki.issn.1674-8697.2024.18.007

[10] Zhang Y, Yang Q, Huang Y, Zeng C, Luo X, Tan Y. A digital data collection study of tombs based on nap-of-the-object photogrammetry. Journal of Nanning Normal University (Natural Science Edition). 2023;40(3):136–42. doi: 10.16601/j.cnki.issn2096-7330.2023.03.021

[11] Xue X, Ma Z, Wang Y, Shi C, Li Z. Hydrogeochemical characteristics of groundwater and its impacts on ancient tombs in Baling Mountain Tomb Group Reserve. Geological Survey of China. 2023;10(4):72–80. doi: 10.19388/j.zgdzdc.2023.04.09

[12] Bie X. Analyzing the value and protection of the Shentou tomb groups. Identification and appreciation to cultural relics. 2024;277(10):44–7. doi: 10.20005/j.cnki.issn.1674-8697.2024.10.011

[13] Huang Y. Investigation of ancient tomb burials in Quanzhou. Journal of Chinese Antiquity. 2019;(2):32–4.

[14] Shen J, Zhang R, Li G. Study on the spatial and temporal distribution characteristics of cultural relics protection units in southern Sichuan. Urbanism and Architecture. 2023;20(2):132–4. doi: 10.19892/j.cnki.csjz.2023.02.37

[15] Zhao C, Huang X, Zhang L. Analysis on temporal and spatial distribution characteristics and influencing factors of cultural heritage in Chengdu. Beijing Surveying and Mapping. 2022;36(5):636–43. doi: 10.19580/j.cnki.1007-3000.2022.05.021

[16] Zhou K, Li Y, Wang P, Chu Z, Wang C, Li A. The spatial - temporal distribution characteristics of cultural relics in Beijing. Journal of Arid Land Resources and Environment. 2024;38(10):70–9. doi: 10.13448/j.cnki.jalre.2024.206

[17] Li J, Hu M, Zhang D, Zhao Y. Spatial distribution characteristics of cultural relics in the Yellow River basin and influencing factors. Journal of Arid Land Resources and Environment. 2021;35(10):194–201. doi: 10.13448/j.cnki.jalre.2021.288

[18] Tu P, Zhou Q. Spatial Distribution Characteristics and Influencing Factors of Fuzhou Place Names Cultural Landscape. Journal of Huaqiao University (Natural Science). 2021;42(2):199–206. doi: 10.11830

[19] Wei H, Wang G. Study on vitality influencing factors of Nanning historical urban area based on geographically and temporally weighted regression model. Design Community. 2024;(4):115–23.

[20] Zhang R, Liu W, Song Z. Spatio-temporal evolution and driving factors of national development zones of China based on geodetectors. JOURNAL OF NATURAL RESOURCES. 2021;36(10):2672. doi: 10.31497/zrzyxb.20211015

[21] Li L, Zhang L, Tan B. The Spatial and Temporal Distribution Characteristics of Cultural Relics Protection Units in Hubei Province. Journal of Xinjiang Normal University (Natural Sciences Edition). 2024. doi: 10.14100/j.cnki.1008-9659.20240923.002

[22] Wu J, Chen Y, Jiao M, Du B. Spatial-Temporal Characteristics and Impacting Factors of Yunnan Cultural Heritages: Based on the Perspective of Key Cultural Relics Protection Units. Journal of Natural Science of Hunan Normal University. 2023;46(5):52–62. doi: 10.7612/j.issn.2096-5281.2023.05.006

[23] Zhang X, Zhang R, Liu B, Dong J. Study on spatial - temporal distribution characteristics and influencing factors of cultural relic protection units in Gansu Province. Resource Development & Market. 2023;39(04):487–94.

[24] Duan Y, Zhang Y. Study on spatial - temporal distribution characteristics and influencing factors of cultural heritage in Shanxi Province taking cultural relics protection units as an example. Resource Development & Market. 2024;40(7):1094–102. doi: 10.3969/j.issn.1005-8141.2024.07.015

[25] Wang Z, Chen S. Spatial pattern and differences of cultural relics conservation units in Guangdong province. Geospatial Information. 2022;20(4). doi: 10.3969/j.issn.1672-4623.2022.04.006

[26] Kang X, Xie X. Temporal and Spatial Distribution Characteristics of Cultural Relic Protection Units and Its Influencing Factors in Qinghai Province. Journal of Subtropical Resources and Environment. 2023;18(4):114–22. doi: 10.19687/j.cnki.1673-7105.2023.04.014

[27] Yang Y, Zhu F. Research on logistics demand forecasting in Guangxi based on ridge regression model. Logistics Sci-Tech. 2024. doi: 10.1373.F.20240729.1432.002

[28] Wang Y. The spatial heterogeneity of the mechanisms shaping intergenerational mobility in China: An empirical study based on a geographically weighted regression model. Zhejiang Social Sciences. 2024;11:87–99. doi: 10.14167/j.zjss.2024.11.008

[29] Zhang X. Characterization of spatial distribution of immovable cultural relics structure in Shanxi Province and analysis of influencing factors. Identification and Appreciation to Cultural Relics. 2017;(8):85–7.

[30] Liang S. A history of Chinese architecture. Beijing: Sanlian Bookstore. 2011.

[31] Chen J, Zhou Y, Liu D. Analysis of spatio —— temporal distribution characteristics of ancient architecture heritage in China. Journal of Arid Land Resources and Environment. 2018;32(2):194–200. doi: 10.13448/j.cnki.jalre.2018.069

[32] Wang F, Jiang W, Li Q, Kong X, Lu L. Temporal and spatial pattern of cultural heritage in Shanxi province and its influencing factors: A case study of cultural relics protection units. Journal of Anhui Normal University (Natural Science). 2024;47(2):161–70. doi: 10.14182/J.cnki.1001-2443.2024.02.009

[33] Su P, Zhou D, Li X. A General History of Bashu Culture: The Historical Scholarship Volume. Chengdu: Sichuan People’s Publishing House. 2021.

[34] Ge J. The Yellow River and Chinese Civilization. Beijing: The Commercial Press. 2023.

[35] Yang H. Studies on Yangtze River Civilization. Wuhan: Changjiang Publishing House. 2021.

[36] Qian M. Gains and losses of political systems in Chinese history. Beijing: Sanlian Bookstore. 2025.

[37] Xu W, Zeng L. Temporal and spatial distribution pattern and forming factors of cultural heritage in Shaanxi Province: Based on the National Key Cultural Relics Protection Units. Journal of Northwest University (Natural Science Edition). 2021;51(3):438–46. doi: 10.16152/j.cnki.xdxbzr.2021-03-012

[38] Xu J, He X. Study on the spatial distribution of cultural relics in Luoyang based on GIS technology. Jiangxi Science. 2017;35(6):878–85. doi: 10.13990/j.issn1001-3679.2017.06.012

